# Pentraxin-3 and Other Inflammatory Markers for an Infected Diabetic Foot Ulcer Diagnosis: A Prospective Study

**DOI:** 10.3390/diagnostics13142366

**Published:** 2023-07-13

**Authors:** Andrei Ardelean, Diana-Federica Balta, Carmen Neamtu, Adriana Andreea Neamtu, Mihai Rosu, Luminita Pilat, Silviu Moldovan, Cristi Tarta, Bogdan Totolici

**Affiliations:** 1 1st Surgery Clinic, Faculty of Medicine, West University “Vasile Goldis” Arad, 310025 Arad, Romania; 2 Faculty of Medicine, West University “Vasile Goldis” Arad, 310025 Arad, Romania; 3 Department X, 2nd Surgical Clinic, Researching Future Chirurgie 2, “Victor Babeș” University of Medicine and Pharmacy, Eftimie Murgu Sq. No. 2, 300041 Timișoara, Romania

**Keywords:** pentraxin-3, diabetic foot ulcer, CRP, limb amputation, diabetes mellitus, procalcitonin, fibrinogen, ESR

## Abstract

Strategies have been researched and implemented to reduce the number of people with diabetic foot ulcers (DFUs). One problem is the accurate assessment of DFU severity, which is the main factor in resource allocation and treatment choice. The primary objective of this study was to assess pentraxin-3 as a biomarker of an infected DFU (IDFU), the limb amputation level prognosis, and patient survival. The secondary objectives were to evaluate and compare other markers, including white blood cells (WBCs), C-reactive protein (CRP), the erythrocyte sedimentation rate (ESR), and procalcitonin (PCT), for identifying IDFUs. Over a period of two years, 145 patients were followed; 131 of these were analyzed for this study. Pentraxin-3 was found to be a good predictor of death (*p* = 0.047). A comparison between IDFUs and DFUs revealed the following differences: PCT had the highest AUROC of 0.91, sensitivity of 93.7, and specificity of 83.3%. CRP had a cutoff value of 226 mg/L, an AUROC of 0.89, a sensitivity of 95.5%, and a specificity of 83.3%. Fibrinogen had an AUROC of 0.87 at a cutoff value of 5.29 g/L, with a good sensitivity and specificity of 85% and 87%, respectively. ESR had a cutoff value of 46 mm/h, an AUROC of 85%, a sensitivity of 83.7%, and a specificity of 83.3%. Pentraxin-3 showed promising results in predicting IDFUs and DFUs, and it served as a marker for the risk of death in IDFU patients during the 6 month follow-up. Other markers, including CRP, PCT, ESR, and fibrinogen, were more effective in differentiating between IDFUs and DFUs.

## 1. Introduction

There is increasing concern worldwide about the increasing number of people with diabetes mellitus (DM), which is expected to reach 600 million by 2035 [1]. The lifetime risk for developing a diabetic foot ulcer (DFU) was assessed as between 15% and 25% [2], although some calculations are more pessimistic and consider that one in three patients with DM will suffer from a DFU during their lifespan [3]. The consequence of having diabetes mellitus is a high glycemia level, which over time will lead to macrovascular and microvascular complications. Some macrovascular complications (coronary artery disease, peripheral arterial disease, and cerebrovascular disease) and some microvascular complications (diabetic retinopathy, nephropathy, and neuropathy) can trigger a DFU through multiple mechanisms. All these factors determine the risk of foot ulcers, which are difficult to heal due to polyneuropathy, foot deformity, lack of a protective sense of pain, reduced eyesight with consecutive foot trauma, poor response to infection, inflammation, and disturbed immunity. It is important to prevent these DFUs by improving patient awareness and education.

The influence of a DFU on a diabetic person’s life expectancy was found to be significant, with a 2.5-fold increased risk of dying after 5 years compared to people with DM and no DFU [4]. The risk of a limb amputation after an infected diabetic foot ulcer (IDFU) after 1 year was found to be up to 17%, while death occurred in 15% of patients [3]. In another study, patients with an IDFU and a limb amputation had a worse life expectancy than many cancers, with up to a 70% death rate after 5 years, and people with an IDFU presenting concomitant end-stage renal disease and peripheral arterial disease (PAD) requiring revascularization had a 5% chance of survival after 5 years [3,5,6].

DFUs have a high rate of recurrence, reaching a staggering 65% after 3 years, which has prompted a reevaluation of the use of the term healing of this disease to preferring the term remission [3]. Due to their prevalence and high recurrence rates, DFUs are among the leading causes of health-related expenditure worldwide. Social and production losses should also be considered for those with limb amputation after DFUs, making DFUs a notable burden.

Strategies have been researched and implemented to reduce the number of people with DFUs, including better methods of diagnosis and treatment of this disease to prevent relapses and limb amputation. One problem with these efforts is the accurate assessment of DFU severity, which is the main factor in resource allocation and treatment algorithm choice. The International Working Group on the Diabetic Foot (IWGDF) has published guidelines to diagnose and treat DFUs [7], but the infection signs are sometimes subtle in a diabetic person, and evolution of the disease could be fulminant; therefore, additional disease severity markers are needed to improve clinical decisions. The most suitable markers for this are inflammatory markers, such as the number of leucocytes (WBCs), erythrocyte sedimentation rate (ESR), fibrinogen, C-reactive protein (CRP), and procalcitonin (PCT), all of which have been researched in many studies [8].

Pentraxin-3 is an acute-phase reactant related to CRP. Both are members of the pentraxin family. Pentraxin-3 has an important role in the antimicrobial response and clearing of cellular debris [9,10]. Produced by various cells involved in the local inflammatory response and local defense against infections, pentraxin-3 might be a better biomarker for IDFU than other systemic markers [10]. Pentraxin-3 has been proven to be related to disease severity and worse outcomes in various diseases such as cancer, myocardial infarction, acute respiratory distress syndrome, and sepsis [10]. Studies have linked pentraxin-3 to DFUs [9] as a marker of soft-tissue necrotizing infections, including different types of limb infections [10].

The primary objective of this study was to assess pentraxin-3 as a biomarker of DFU infection, limb amputation level prognosis, and patient survival. The secondary objectives were to evaluate and compare other markers, including WBCs, CRP, ESR, PCT, glycosylated hemoglobin (Hba1c), hemoglobin, and duration of diabetes, in relation to the prognosis of DFUs.

## 2. Materials and Methods

### 2.1. Patient Selection

This was a single-center prospective study at Arad County Emergency Hospital between March 2020 and March 2022. Patients were recruited, were informed about the nature of our study, and signed an informed consent form before their inclusion. There were three groups of patients enrolled: group A included 90 patients with IDFU, group B had 30 patients with DFUs but no infection, and group C comprised 25 patients as a control group of healthy subjects with no acute or chronic inflammatory disease, matched for age and sex with the other two groups. Patients with an IDFU were all admitted to our unit in line with the general practice of our health system. The classification system for defining the presence and severity of an infection of the foot in a person with diabetes proposed by the IWGDF in 2019 was used [7]. Group A was further analyzed on the basis of its subgroups, with healed patients, patients who needed other procedures, and dead patients.

Surgical procedures used were classified as follows: (1) surgical debridement of the soft tissue without amputation of the bone; (2) toe amputation; (3) transmetatarsal amputation; (4) midtarsal amputation; (5) transtibial amputation, i.e., below-the-knee amputation; and (6) transfemural amputation, i.e., above-the-knee amputation [11].

### 2.2. Patient Charatceristics: Inclusion and Excluzion Criteria

The inclusion criteria were as follows: (1) age above 18; (2) ability to understand and sign an informed consent form; (3) a signed informed consent form; (4) an IDFU with the index presentation at our hospital with no prior surgical or antibiotic therapy; (5) a mild or moderate IDFU according to the IWGDF classification [7]; and (6) a positive wound microbiology culture.

The following exclusion criteria were set to minimize sources of biases: (1) other infections; (2) death by COVID-19 during the study period or follow-up; (3) other concomitant inflammatory or infectious disease for the control group; (4) malignancy discovered before or during the study period; (5) loss of follow-up at 6 months; and (6) indication for major vascular reconstructive procedures.

### 2.3. Ethics

Ethical approval was obtained from the Arad County Hospital Ethics Committee by the investigators (approval number 51; 24 February 2020), and the study was conducted according to the Declaration of Helsinki. All patients signed an informed consent form to allow the collection and maintenance of their demographic data, blood tests, imagistic tests, and wound data.

### 2.4. Data Collection

Age, rural or urban habitation, sex, other comorbidities, site of the infection, types of amputation, and hospital stay were retrieved from the patients’ file charts. The decision for amputation and the level of the amputation were based on a proper assessment of the patient during our clinic daily meetings, and this was seen as a last resort. Standard laboratory blood tests were performed: WBCs (number/mL), ESR (mm/h), thrombocytes (number/mL), HbA1c (%), hemoglobin (g/dL), and fibrinogen (mg/dL). All data were collected from the index admission.

Pentraxin-3 kits were purchased from MyBioSource, Inc. (San Diego, CA 92195-3308, USA) and were manipulated according to the manufacturer’s manual recommendations (see https://www.mybiosource.com/ptx3-human-elisa-kits/pentraxin-3/26553, accessed on 20 February 2020).

### 2.5. Follow-Up

The final follow-up was 6 months after index admission and consisted of a visit to our outpatient clinic. If the patients presented to our hospital during this period, these visits or readmissions were also recorded. Deaths were recorded using the local death registry. Other surgeries or procedures were also recorded at 6 months.

### 2.6. Statistical Analysis

The statistical analysis was performed using SPSS, Version 27.0 (IBM Statistics), and MS Excel, Office 2019 (Microsoft). The distribution of numerical variables was assessed using the Kolmogorov–Smirnov test, and numerical variables with a Gaussian distribution were presented as the mean value and standard deviation or error, while variables with a non-Gaussian distribution were presented as median values and range intervals. A *t*-test was used to compare continuous variables with a Gaussian distribution, and the Mann–Whitney U test was used in the case of variables with a non-Gaussian distribution. Group comparisons of categorical variables were performed using the chi-square test. The Pearson (*r*) and Spearman (rho) correlation coefficients were used to establish correlations between continuous variables and evaluate monotonic relationships, respectively; a *p*-value < 0.05 was considered to indicate statistical significance.

## 3. Results

**Figure 1:**
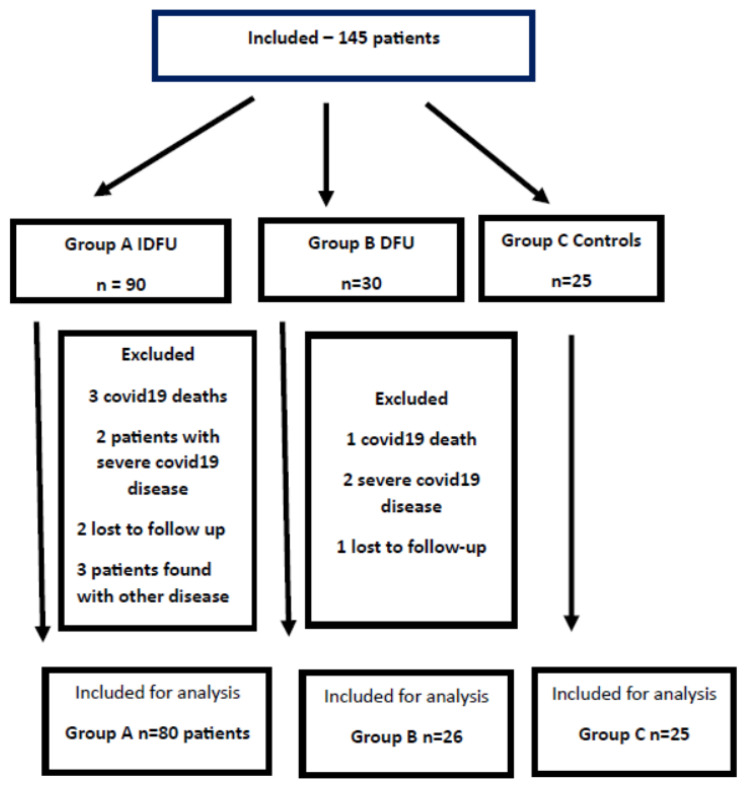
Flowchart of patient enrolment in the study.

There were 145 patients included in the study: 90 with IDFU, 30 with DFU, and 25 controls. Figure 1 shows the flowchart of patient inclusions and exclusions.

We found differences among the three groups in several inflammatory markers, as shown in Table 1.

When trying to discriminate between an IDFU and a DFU, groups A and B, some markers achieved a good statistical result. We calculated the cutoff values and different statistical variables, and the best predictors were CRP, fibrinogen, and ESR according to their AUROC (area under the curve), as shown in Table 2.

**Figure 2:**
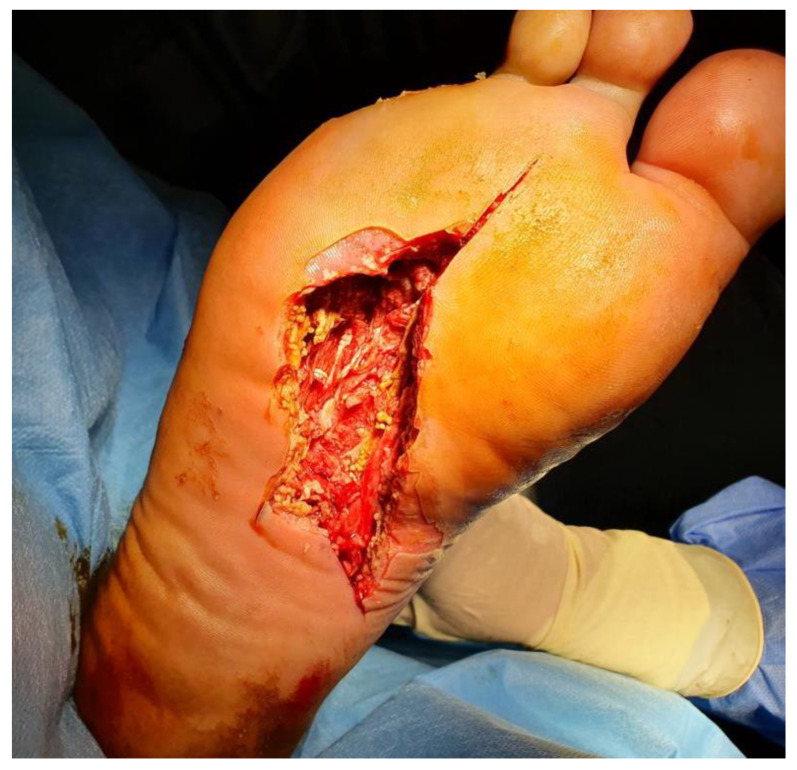
Infected diabetic foot ulcer on the plantar aspect of the foot: debridement including plantar fascia; recurrence after toe amputation.

**Figure 3:**
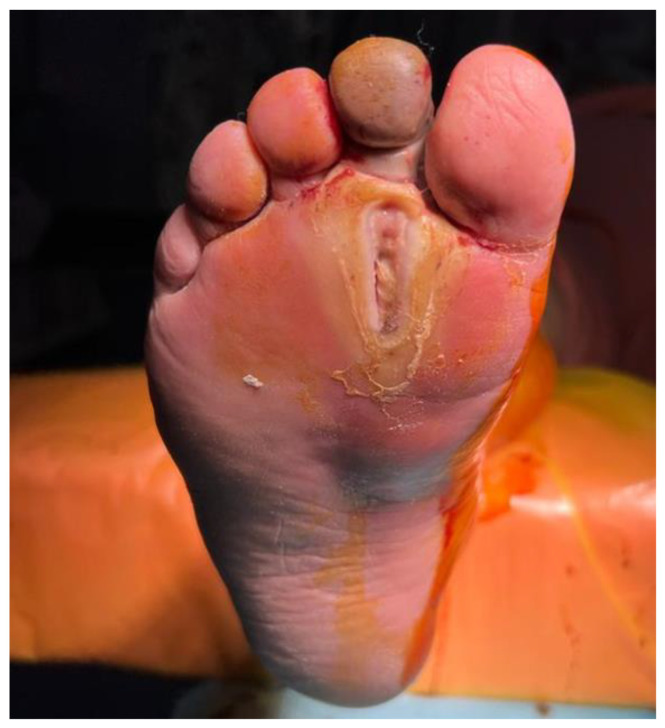
Infected diabetic foot ulcer with infection along the plantar fascia and abscess at this level.

**Figure 4:**
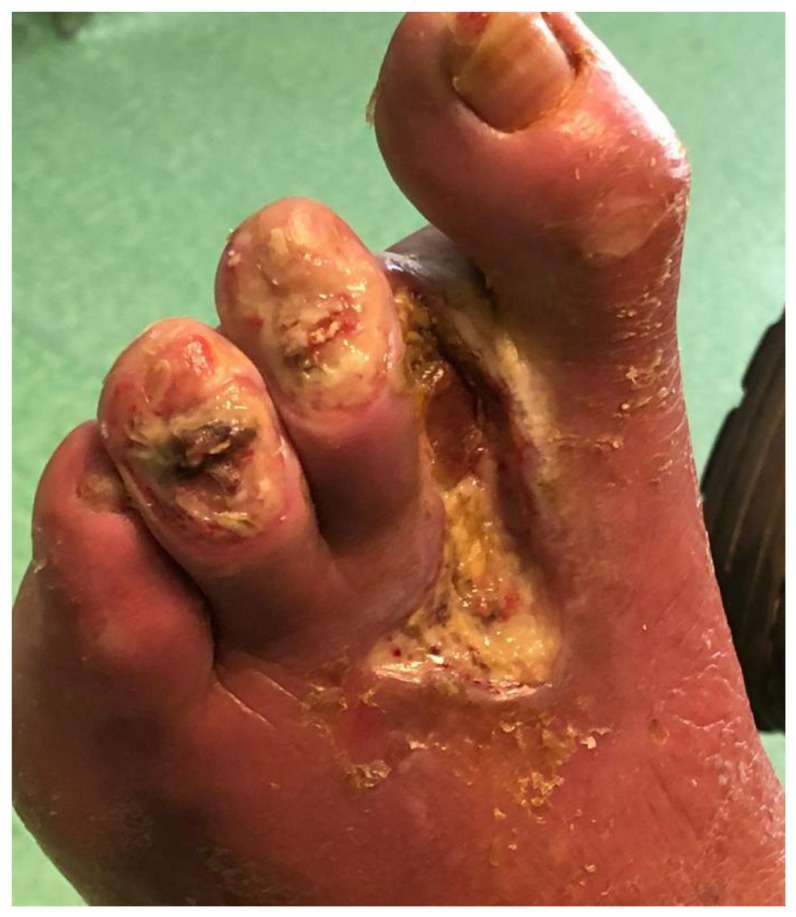
Recurrent infected diabetic foot ulcer with previous toe amputation.

Analyzing the two subgroups of group A regarding IDFUs, based on the level of surgery, i.e., distal (*n* = 63) to the ankle, including debridement (Figure 2 and Figure 3) and toe or foot amputation (Figure 4), or proximal (*n* = 17) to the ankle, we found the outcomes listed in Table 3.

Table 4 shows a comparison of three subgroups of group A: survivors with no further amputation until FU, survivors with other amputation at 6 months, and deceased patients.

The most important risk factor for mortality was found to be procalcitonin, with an OR of 2.868. In differentiating among IDFUs, DFUs, and controls, the best options were ESR, PCR, PCT, HbA1c, pentraxin-3, and hemoglobin. Regarding the prediction of distal or proximal amputation, only WBCs and HbA1c were good predictors.

## 4. Discussion

Pentraxin-3 was found to be a predictor of mortality and reoperation in patients with IDFUs. This was the same outcome found by Bastrup-Birk et al. in a larger study of patients with heart disease [12], although the values of PTX3 were lower in our study. Hansen et al. did not find a relationship between PTX3 levels and the death of patients with necrotizing tissue infections at 6 months [10]. PTX3 was significantly higher (*p* = 0.003) in patients with IDFUs compared to DFU patients and heathy controls; however, when we analyzed the AUROC, it reached a poor value of 0.62, with a specificity less than 50%. This could be due to the fact that PTX3 has been demonstrated as a marker of vascular disease and metabolic syndrome [13,14], which are presented by the majority of patients with diabetes. Balin et al. found lower levels of PTX3 as a predictor for infection and amputation compared to controls [9]. PTX3 failed to discriminate between the limb amputation level distal and proximal to the ankle; however, in our study, it showed higher values in distal limb surgeries, albeit without statistical significance. Hansen et al. found a relationship between PTX3 and the need for limb amputation in a subgroup of their study [10].

ESR is a marker of general inflammation; in our study, ESR proved to be a good marker for differentiating among IDFUs, DFUs, and controls (*p* = 0.001), with a cutoff value of 46 mm/h. It had a good AUROC value of 85%, a sensitivity of 83.7%, a specificity of 83.3%, a positive likelihood ratio of 5.63, and a negative likelihood ratio of 0.20. These values were similar to those found by Sharma et al. in their latest review of inflammatory markers in IDFUs, with an AUROC of 88.5%, a sensitivity of 72%, a specificity of 76%, an LR+ value of 4.61, and an LR− value of 0.278. ESR could be a useful, simple, and easily available marker for IDFUs when deciding on the admission of a patient and antibiotic therapy [8].

Fibrinogen was another acute phase inflammatory marker proven to be useful in differentiating IDFUs from DFUs, with an AUROC of 0.87 at a cutoff value of 5.29 g/L, with a good sensitivity and specificity of 85% and 87%, respectively, the highest LR+ of 6.8 of all markers, and an LR− of 0.10. In other studies, fibrinogen showed an excellent AUROC of 0.941 [15] and 0.858 [16], with better sensitivity and specificity than in our study. Li et al. found that fibrinogen was a marker that could predict different types of lower limb amputations, and the cutoff value for this was 5.67 g/L, similar to the value in our study, in which 67 patients underwent different types or levels of amputation [16]. Fibrinogen levels can rise in many inflammatory or infectious diseases, and these concomitant conditions have to be ruled out when diagnosing an IDFU as the single cause of increased fibrinogen.

CRP has proven to be one of the most reliable markers for many infectious or inflammatory diseases, and IDFUs are no exception. Our statistical values matched the literature for CRP, with a cutoff value of 226 mg/L, an AUROC of 0.89, a sensitivity of 95.5%, a specificity of 83.3%, an LR+ of 5.85, and an LR− of 0.03, which were close to those found in the review of Sharma et al., with a cutoff value of 225.1 mg/L, an AUROC of 0.89, a sensitivity of 77.4%, a specificity of 84.3%, an LR+ of 5.08, and an LR− of 0.26 [8]. Our study was conducted throughout the SARS-CoV-2 pandemic, and we excluded patients with severe forms of COVID-19; however, we were not able to exclude all positive patients. Thus, this may have been a source of bias when interpreting the value of CRP, which is elevated in COVID-19 patients [17].

Procalcitonin is a polypeptide protein secreted by several organs, including thyroid C-cells and the liver, lungs, and renal parenchymal cells. PCT plays an important role in diagnosing bacterial infections in the body. PCT is a sensitive biomarker of local infections, generalized inflammation, and sepsis. PCT has been evaluated to diagnose and to differentiate an IDFU and its grades [15,18] with very accurate results, but different cutoff values. When differentiating between IDFUs and DFUs, we found the highest AUROC for PCT (0.91), with a sensitivity of 93.7%, a specificity of 83.3%, an LR+ of 5.86, and an LR− of 0.07. The cutoff value was 0.26 mg/dL, which was the same cutoff value obtained in a review by Majeed et al. [19]. Umapathy et al. found that a cut-off value of 0.5 mg/dL had an AUROC of 99%, with a 54% sensitivity and a 100% specificity for PCT with a positive predictive value of 100% and a negative predictive value of 12% for IDFU diagnosis [20].

The presence of a DFU was associated with death of the patient as an independent variable, regardless of age, gender, diabetes mellitus complication count, physical or visual impairment, and diabetes mellitus duration, reaching a 14% death rate after 3 years. The majority of deaths were caused by infections (in one-third of the patients) [21]. The death rate after limb amputation varied across studies depending on the population screened and the level of amputation [22]. Jones et al. reported, in a massive study which included 186,338 patients over 8 years, that the 30 day, 1 year, and 3 year overall mortality rates after major lower limb amputation were 13.5%, 48.3%, and 70.9%, respectively [23]. Fortington et al. found death rates up to 22% after 30 days, 44% after 1 year, and 77% after 5 years, but all these studies included only major amputations [22]. Even though we had a more heterogenous population of patients in our study, with only 21.25% major amputations, we had a 12.5% death rate at 6 months. Half of these patients had major amputations, while the other half had minor amputations and could walk after surgery. This lower rate of death could also be explained by the lower age of our patients compared with other studies, at 64 years versus 74 years [22] or 77 years [23]. Kristensen et al., in a similar series, reported differences in mortality related to the level of amputation, as well as the number of comorbidities, but a very high 30 day death rate of 30% was maintained after major amputations [24]. Some authors have suggested that an episode of a DFU is just the early sign of a major future cardiovascular event which will threaten the life of the patient, with multiple local pathways affecting tissue integrity being replicated in the whole body with the progression of micro- and macro-vasculopathy, in conjunction with neuropathy and chronic inflammation [25,26]. In an Australian study, the authors found that people with diabetes mellitus and an IDFU died at least 15 years earlier than their fellow citizens [27]. The most important risk factor for mortality in our study was found to be procalcitonin levels, with an OR of 2.868. Pentraxin-3 showed a statistically significant difference between survivors and non-survivors; this may be due to the general inflammatory state of people with diabetes [28] and could be associated with infection and consecutive sepsis in some IDFU patients, given that pentraxin-3 is a known marker of sepsis [29].

WBCs were found to be a good predictor of IDFUs, but it should be kept in mind that WBCs increase regardless of the inflammation or infection occurring in the body. With an AUROC of 0.79, a sensitivity of 87.5%, and a specificity of 62.5%, we found numbers similar to those in the literature [8].

We did not find any influence of diabetes mellitus duration on the prognosis of IDFUs in our study. Previous studies have shown an increased risk of DFUs related to diabetes mellitus duration and insulin therapy duration [30].

These markers all showed promising results in differentiating IDFUs from DFUs; as IDFUs are a disease which may enter remission rather than one that is healed, some of these markers could be researched as possible markers of remission and recurrence.

Researchers have been interested in finding the differences between IDFUs and DFUs, and these markers may be suitable for prognostic purposes [31]. Our objective was to identify the differences between above- and below-the-ankle limb amputation, and we found that only WBCs and HbA1c showed significant differences. HbA1c levels were lower in cases requiring proximal amputation, indicating more severe forms of the disease. Higher levels of HbA1c were related to a worse prognosis for IDFUs in a study by Lee et al. [32]. In a select group of patients with IDFUs, a lower HbA1c at baseline could predict an above- or below-the-knee amputation. Yazdan et al. found a higher HbA1c to be a risk for developing a DFU [29]. Higher HbA1c is a risk factor for DFUs or IDFUs; however, once an IDFU develops, a lower HbA1c value was a negative prognostic indicator, which could be linked to the nutritional status or general frailty of these patients due to age and comorbidities associated with diabetes mellitus, inflammation, and infection [33].

Several markers were prognostic for reoperation, even though these results should be interpreted with caution, as people with more severe disease and proximal limb amputation all entered the healed group or death group. Nonetheless, PCT, pentraxin-3, and the hospital stay were significantly higher in the death group.

One potential source of bias in our study was the COVID-19 pandemic period. We made efforts to exclude patients affected by the virus and to focus on the true cause of death or the cause of the inflammatory response, considering that diabetic and frail patients are at higher risk of mortality from the virus. Other sources of bias included the timeline for collecting blood samples, which was reported as the disease onset, as different inflammatory markers peak at different times after disease onset. One strength of our study was the completion of follow-up for all enrolled patients.

## 5. Conclusions

Pentraxin-3 showed promising results in predicting IDFUs and DFUs, and it served as a marker for the risk of death in IDFU patients during the 6 month follow-up period. Other markers, including CRP, PCT, ESR, and fibrinogen, were more effective in differentiating between IDFUs and DFUs.

## Figures and Tables

**Table 1:** Comparison of demographics and inflammatory markers between the three groups.

Variables/Group	A (*n* = 80)	B (*n* = 26)	C (*n* = 25)	*p*-Value
Age	64.35 ± 10.38	60 ± 12.16	58 ± 14.11	0.785
Sex, male %	67.50	53.84	60	0.508
Urban, %	38.80	44.00	34.80	0.734
WBCs (number × 10^9^/L)	15.66 ± 6.01	10.69 ± 6.47	7.54 ± 3.31	0.05
ESR (mm/h)	61.80 ± 21.36	26.96 ± 19.48	16 ± 16.37	0.001
Fibrinogen (mg/dL)	652.69 ± 190.05	442.39 ± 152.16	336.28 ± 120.46	0.086
CRP (mg/dL)	114.25 ± 100.34	33.34 ± 81.77	3.25 ± 7.47	0.001
PCT (ng/mL)	0.71 ± 0.59	0.19 ± 0.53	0.04 ± 0.02	0.006
Pentraxin-3 (pg/mL)	3262.26 ± 1624.89	1975.22 ± 853.98	1149.61 ± 481.73	0.003
HbA1c (%)	8.64 ± 2.89	9.25 ± 2.51	5.36 ± 0.26	0.001
Hemoglobin (g/dL)	11.22 ± 2.15	13.5 ± 2.21	14.13 ± 1.66	0.001
Thrombocytes (number × 10^9^/L)	356.04 ± 143.32	251.30 ± 84.74	240.16 ± 51.36	0.840

HbA1c = glycosylated hemoglobin, WBCs = white blood cells, CRP = C-reactive protein, PCT = procalcitonin, ESR = erythrocyte sedimentation rate.

**Table 2:** Inflammatory markers differentiating between group A (IDFU) and group B (DFU).

Variable	Cutoff Value	AUROC	*p*-Value	Se	Sp	PPV	NPV	LR+	LR−
Fibrinogen (mg/dL)	>529	0.87 (0.78–0.92)	<0.0001	85	87	95.8	63.6	6.8	0.10
HbA1c (%)	<8.7	0.5(0.4–0.6)	0.8	55	54.2	79	26.5	1.2	0.30
Hemoglobin (g/dL)	<13.2	0.73 (0.6–0.8)	0.0002	80	62.5	87.7	48.4	2.13	0.30
WBCs (number × 10^9^/L)	>9.64	0.79 (0.7–0.8)	<0.0001	87.5	62.5	88.6	60	2.33	0.20
CRP (mg/dL)	>22.6	0.89 (0.8–0.9)	<0.0001	95.5	83.3	95.1	89.6	5.85	0.03
Pentraxin-3 (pg/mL)	>2372	0.62 (0.54–0.65)	0.47	82.4	45.8	84.0	81.2	1.85	0.50
PCT (ng/mL)	>0.28	0.91 (0.5–0.7)	<0.0001	93.7	83.3	94.9	80	5.63	0.07
ESR (mm/h)	>46	0.85 (0.7–0.9)	<0.0001	83.7	83.3	94.4	60.6	5.03	0.20

HbA1c = glycosylated hemoglobin, WBCs = white blood cells, CRP = C-reactive protein, PCT = procalcitonin, ESR = erythrocyte sedimentation rate, AUROC = area under the curve; Se = sensibility; Sp = specificity; PPV = positive predictive value, NPV = negative predictive value, LR+ = positive likelihood ratio; LR− = negative likelihood ratio.

**Table 3:** Distal versus proximal surgery in group A.

Variables/Groups	A Distal (*n* = 63)	A Proximal (*n* = 17)	*p*-Value
Age, years	63.76 ± 10.40	66.53 ± 10.30	0.332
Male, %	68.30	64.70	0.785
Urban, %	38.10	41.20	0.820
Hospital stays, days	8 (3–32)	10 (3–26)	0.001
WBCs (number × 10^9^/L)	14.78 ± 5.57	18.92 ± 6.63	0.011
CRP (mg/dL)	105.77 ± 105.80	145.64 ± 70.73	0.147
PCT (ng/mL)	0.69 ± 0.37	0.98 ± 0.54	0.339
Pentraxin-3 (pg/mL)	4372.02 ± 1650.29	2855.51 ± 1503.25	0.247
HbA1c (%)	9.00 ± 2.78	7.28 ± 2.96	0.028
Diabetes history, %			
5 years 5–10 years 10 years	23.80 38.10 38.10	23.50 17.60 58.80	0.338
6 month FU			
Healed Other amputations Death	55.60 31.70 12.70	76.50 11.80 11.80	0.090

HbA1c = glycosylated hemoglobin, WBCs = white blood cells, CRP = C-reactive protein, PCT = procalcitonin, ESR = erythrocyte sedimentation rate, FU = follow-up.

**Table 4:** Comparison of three subgroups of group A: survivors with no further amputation until FU, survivors with other amputation at 6 months, and deceased patients.

Variables/Groups	Survival Healed (*n* = 48)	Survival with New Amputation (*n* = 22)	Death (*n* = 10)	*p*-Value
Age, years	64.79 ± 11.41	63.59 ± 7.74	63.90 ± 11.10	0.820
Male, %	66.70	77.30	50.00	0.327
Urban, %	39.60	40.90	30.00	0.578
Surgery, % 1 2 3 4 5 6	10.40 52.10 8.30 2.10 12.50 12.50 2.10	18.20 54.50 13.60 4.50 9.10 0 0	20.00 10.00 10.00 40.00 0 10.00 10.00	0.332
Hospital stay, days	8 (3–26)	7 (3–23)	8 (3–32)	0.003
WBCs (number × 10^9^/L)	15.34 ± 5.78	16.08 ± 6.03	16.26 ± 7.50	0.664
ESR (mm/h)	62.33 ± 19.98	56.45 ± 24.90	71.00 ± 17.59	0.209
Fibrinogen (mg/dL)	640.04 ± 233.31	678.05 ± 136.31	657.60 ± 133.14	0.819
CRP (mg/dL)	111.20 ± 109.58	100.32 ± 85.79	159.52 ± 75.44	0.190
PCT (ng/mL)	0.40 ± 0.68	0.23 ± 0.26	1.02 ± 0.89	0.015
Pentraxin-3 (pg/mL)	2283.30 ± 1647.39	3029.43 ± 1881.56	4013.60 ± 1832.38	0.047
HbA1c (%)	8.12 ± 2.95	8.92 ± 2.38	10.48 ± 3.04	0.026
Diabetes history, %				
<5 years 5–10 years >10 years	29.20 29.20 41.70	22.70 36.40 40.90	0 50.00 50.00	0.182

HbA1c = glycosylated hemoglobin, WBCs = white blood cells, CRP = C-reactive protein, PCT = procalcitonin, ESR = erythrocyte sedimentation rate.

## Data Availability

Data are available on request.
